# Homogeneity and heterogeneity in amylase production by *Bacillus subtilis* under different growth conditions

**DOI:** 10.1186/s12934-016-0455-1

**Published:** 2016-03-29

**Authors:** Tina N. Ploss, Ewoud Reilman, Carmine G. Monteferrante, Emma L. Denham, Sjouke Piersma, Anja Lingner, Jari Vehmaanperä, Patrick Lorenz, Jan Maarten van Dijl

**Affiliations:** AB Enzymes GmbH, Feldbergstrasse 78, 64293 Darmstadt, Germany; Department of Medical Microbiology, University of Groningen, University Medical Center Groningen, Hanzeplein 1, 9700 RD Groningen, The Netherlands; Roal Oy, Tykkimäentie 15b, 05200 Rajamäki, Finland; Department of Medical Microbiology and Infectious Diseases, Erasmus University Medical Center Rotterdam, Rotterdam, The Netherlands; Division of Biomedical Sciences, Warwick Medical School, University of Warwick, Coventry, CV4 7AL UK; c-LEcta GmbH, Perlickstraße 5, 04103 Leipzig, Germany

**Keywords:** *Bacillus subtilis*, Amylase, Heterogeneity, Secretion stress, Transcription, Translation

## Abstract

**Background:**

*Bacillus subtilis* is an important cell factory for the biotechnological industry due to its ability to secrete commercially relevant proteins in large amounts directly into the growth medium. However, hyper-secretion of proteins, such as α-amylases, leads to induction of the secretion stress-responsive CssR-CssS regulatory system, resulting in up-regulation of the HtrA and HtrB proteases. These proteases degrade misfolded proteins secreted via the Sec pathway, resulting in a loss of product. The aim of this study was to investigate the secretion stress response in *B. subtilis* 168 cells overproducing the industrially relevant α-amylase AmyM from *Geobacillus stearothermophilus*, which was expressed from the strong promoter *P(amyQ)*-*M*.

**Results:**

Here we show that activity of the *htrB* promoter as induced by overproduction of AmyM was “noisy”, which is indicative for heterogeneous activation of the secretion stress pathway. Plasmids were constructed to allow real-time analysis of *P(amyQ)*-*M* promoter activity and AmyM production by, respectively, transcriptional and out-of-frame translationally coupled fusions with *gfpmut3.* Our results show the emergence of distinct sub-populations of high- and low-level AmyM-producing cells, reflecting heterogeneity in the activity of *P(amyQ)*-*M*. This most likely explains the heterogeneous secretion stress response. Importantly, more homogenous cell populations with regard to *P(amyQ)*-*M* activity were observed for the *B. subtilis* mutant strain 168*degUhy3*2, and the wild-type strain 168 under optimized growth conditions.

**Conclusion:**

Expression heterogeneity of secretory proteins in *B. subtilis* can be suppressed by *degU* mutation and optimized growth conditions. Further, the out-of-frame translational fusion of a gene for a secreted target protein and *gfp* represents a versatile tool for real-time monitoring of protein production and opens novel avenues for *Bacillus* production strain improvement.

## Background

The rod-shaped Gram-positive bacterium *Bacillus subtilis* has a long history of safe use as a production host in the biotechnological industry. It has been implemented for the synthesis of various different products such as proteins, vitamins and antibiotics. Next to *B. licheniformis* and *B. amyloliquefaciens*, *B. subtilis* has become one of the most well-established and relevant workhorses in biotechnology, especially for the production of secreted proteins like proteases and α-amylases [1–3]. Importantly, *B. subtilis* is free of endotoxins and considered suitable for the qualified presumption of safety (QPS) status of the European food safety authority. Accordingly, many *B. subtilis* products have received the generally regarded as safe (GRAS) status of the US Food and Drug Administration. In addition, high-quality genomic sequences, as for *B. subtilis* 168 [4, 5], and well-established protocols for genetic modification [6–9] highly facilitate the construction of improved production hosts.

The ability of *Bacillus* species to secrete high amounts of proteins (up to 20–25 g/l) directly into the fermentation broth is facilitated by its single-membrane physiology. The high secretion capacity of *Bacillus* offers clear advantages for downstream processing and final purification of the target protein [3]. The Sec pathway constitutes the main secretion pathway in *B. subtilis* with ~300 endogenous proteins appearing to be translocated through the cell membrane via this pathway [10–12]. Despite this relatively efficient protein translocation machinery, the secretion yield of most heterologous proteins expressed in *Bacillus* is usually lower than the afore-mentioned 20–25 g/l, imposing economic challenges to the industry. This problem, especially evident for proteins derived from organisms not closely related to *Bacillus*, can be attributed to different bottlenecks in the secretion process. These include poor membrane targeting, inefficient membrane translocation or cell wall passage, slow or incorrect folding of the Sec-dependent exoprotein by PrsA and degradation by proteases [13, 14]. The Sec machinery exclusively transports polypeptides in an unfolded state. Consequently, while exiting the Sec channel at the extra cytoplasmic side of the membrane, the transported proteins have to fold rapidly into their correct structural conformation in order to become active and to achieve stability against proteolytic degradation. Misfolded proteins lead to a cellular stress response that generally results in either refolding or degradation of the affected proteins [15]. Protein secretion stress in *B. subtilis* is usually defined as the stress that induces the two-component regulatory system CssR-CssS [16]. High-level production of Sec-dependent secreted proteins, such as the α-amylase AmyQ from *B. amyloliquefaciens,* leads to an accumulation of misfolded protein at the membrane-cell wall interface, resulting in the activation of the response regulator CssR by phosphorylation [16]. This in turn activates the transcription of *htrA* and *htrB* encoding the membrane-bound proteases HtrA and HtrB, which are responsible for proteolytic cleavage and degradation of misfolded secreted proteins [17, 18]. Previously, it has been shown that the expression level of *htrB* correlates with the level of AmyQ production in *B. subtilis* [19]. However, studies dealing with other secretory proteins, such as lipase A of *B. subtilis* and human interleukin-3, showed that the intensity of the protein-secretion stress response only partly reflected the protein production levels [20]. This implies that induction of the secretion stress response largely depends on the nature of the secreted protein that is overproduced.

For industrial protein production, the question whether target gene expression is homogeneous or heterogeneous is highly relevant [21]. Clearly, to obtain the highest yields possible, homogeneous high-level target gene-expressing populations are most desirable. However, the expression levels of individual genes in a bacterial population are often noisy or heterogeneous, and this applies also to *B. subtilis* [22–25]. The presence of low-expressing cells can thus affect the overall protein yield. In more extreme situations, the population can even be bimodal, in which case expression of the protein of interest depends on a particular sub-population [21, 26].

In the present study, we investigated the induction of the protein secretion stress response in *B. subtilis* 168 upon overproduction of AmyM, an industrially relevant α-amylase from *Geobacillus stearothermophilus* [27–29]. To assess the secretion stress response in detail, the transcriptional activity of the *htrB* promoter *P(htrB)* was analyzed using a promoter-*gfp* fusion. In particular, we investigated the correlation between a heterogeneous protein secretion stress response and expression heterogeneity in *B. subtilis* cells producing AmyM where high-level *amyM* expression was directed by the *P(amyQ)*-*M* promoter. Our results show how a particular mutation in the transcriptional regulator *degU,* as well as the selected growth conditions impact on the heterogeneity of *P(amyQ)*-*M* activity and production of the α-amylase AmyM in *B. subtilis.*

## Methods

### Plasmids, primers bacterial strains and growth conditions

Plasmids and primers used and constructed in this study are described in Tables 1 and 2, respectively. The *B. subtilis* prototype strain 168 and derivatives were transformed as described previously [30]. *B. subtilis* DB104 was used for plasmid construction using standard techniques [31]. Strains used and constructed in this study are listed in Table 3. Lysogeny Broth (LB) was used to grow *B. subtilis* DB104, 168 and derivatives thereof. Live cell array (LCA) experiments were performed by growing *B. subtilis* 168 and derivatives in LB, or EnPressoB medium (BioSilta; note that EnPressoB replaces the previous EnBase Flo medium of the supplier) [32]. Media were supplemented with antibiotics as required in the following concentrations: 10 µg ml^−1^ kanamycin (Km), 3 µg ml^−1^ chloramphenicol (Cm), 5 µg ml^−1^ tetracycline (Tc) or spectinomycin (Sp) 100 µg ml^−1^.

**Table 1:** Plasmids used and constructed in this study

Plasmids	Description	Source of reference
pKTH10	*kan, amyQ*; pUB110	[33]
pDAM	*kan, amyM*	AB Enzymes GmbH
pHB201 2.4	*Cm, ermC, gfp*	AB Enzymes GmbH
pKVM2	*tetM; E. coli*-*Bacillus shuttle vector*	AB Enzymes GmbH
pBaSysBioII	*spec, amp, gfpmut3*	[34]
pDAPamy-gfp	*kan; transcriptional P(amy)*-*gfp fusion*	This study
pDAPamy-gfp tet	*tetM; transcriptional P(amy)*-*gfp fusion*	This study
pDAamyM-gfp	*kan; out*-*of*-*frame translational amyM*-*gfp fusion*	This study

**Table 2:** Primers used in this study

Primers	Sequence (5´–3´)
P amy OV rv	TTCTTCTCCTTTACGCATGTTTCCTCTCCCTCTCATTTTC
gfp OV fw	ATGCGTAAAGGAGAAGAACTTTTCACTG
gfp OV rv2	CTTTTTTTGTCCATTTCTCTTATTTGTATAGTTCATCCATGCC
gfp OV fw 3	CAATGAGAAAAGGAGAAGAACTTTTCACTG
M gfp fw	GAGAAATGGACAAAAAAAGCAAAGGGTTC
M gfp rv3	TTCTTCTCCTTTTCTCATTGTTGCCACGTAACAGTAATG
tetM OV fw	TTAATACTAGTTCACTAAGTTATTTTATTGAACATATATCG
tetM OV rv	TCTGAAAAGGGAATGAAAATTATTAATATTGGAGTTTTAGCTC
KM OV fw2	AATAAAATAACTTAGTGAACTAGTATTAATCTGTTCAGCAATC
KM OV rv2	TAATTTTCATTCCCTTTTCAGATAATTTTAGATTTGC

**Table 3:** Bacterial strains used and generated in this study

Bacterial strains	Relevant properties	Source of reference
*B. subtilis* strains		
168	*trpC2*	UMCG, laboratory stock
168 HmB C5	*trpC2 P(htrB)*-*gfp::spc*	[35]
168 *∆rok*	*trpC2, ∆rok::cm*	UMCG, laboratory stock
168*degUhy32*	*trpC2, degUhy32::kan*	UMCG, laboratory stock
DB104	*His nprR2 nprE18 ∆aprA3*	[36]
168 HmB C5::pKTH10	168 HmB C5 carrying plasmid pKTH10	This study
168 HmB C5::pDAM	168 HmB C5 carrying plasmid pDAM	This study
168::pDAPamy-gfp	168 carrying plasmid pDAPamy-gfp	This study
168::pDAPamy-gfp tet	168 carrying plasmid pDAPamy-gfp tet	This study
168*degUhy32*::pDAPamy-gfp tet	168*degUhy32* carrying plasmid pDAPamy-gfp tet	This study
168*∆rok*::pDAPamy-gfp tet	168*∆rok* carrying plasmid pDAPamy-gfp tet	This study
168::pDAamyM-gfp	168 carrying plasmid pDAamyM-gfp	This study

#### Polymerase chain reaction (PCR)

AccuPrime**™** Pfx DNA Polymerase (Life Technologies) was used for DNA amplification by PCR following manufacturer’s instructions.

### Construction of a transcriptional fusion of the *P(amyQ)*-*M* promoter with *gfp*

#### Plasmid pDAPamy-gfp

For monitoring the promoter activity of *P(amyQ)*-*M* from *B. amyloliquefaciens* and heterogeneous or homogeneous activity of *P(amyQ)*-*M* in *B. subtilis* 168, the high-copy plasmid pDAPamy-gfp was constructed by amplifying the high-copy number vector pDAM carrying the *P(amyQ)*-*M* promoter and the *gfpmut3* gene from plasmid pHB201 2.4 and introducing complementary sequences to the 3´-ends with a Tm >50 °C. Plasmid pHB201 2.4 contains the *gfpmut3* gene derived from vector pBaSysBioII. *gfpmut3* was amplified from the ATG start codon with primers gfp OV fw and gfp OV rv2 resulting in the insertion of a complementary sequence to the vector pDAM at the 3´-end. A truncated version of the vector pDAM was amplified using the primers Pamy OV rv and M gfp fw by adding sequences complementary to the *gfpmut3* gene. Amplified products were purified using the Wizard SV gel purification Kit (Promega) and were fused by using the Gibson Assembly Kit (New England Biolabs). The product was directly used to transform competent cells of the intermediate host *B. subtilis* DB104.

#### Plasmid pDAPamy-gfp tet

The kanamycin resistance gene (*kan*) in plasmid pDAPamy-gfp was replaced by the *tetM* gene from pKVM2. The complete vector pDAPamy-gfp, lacking the *kan* gene, was amplified using oligonucleotides KM OV fw2 and KM OV rv2 and inserting ends complementary to the *tetM* gene. The *tetM* gene from pKVM2 was amplified by PCR with primers tetM OV fw and tetM OV rv. Amplified products purified by gel extraction were fused by Gibson Assembly and used to transform competent cells of DB104.

### Construction of translational fusions of the α-amylase *amyM* gene with *gfp*

For real-time monitoring of translation levels of the secreted α-amylase AmyM from *G. stearothermophilus*, the *gfpmut3* gene from pHB201 2.4 was fused to the 3´-end of *amyM* such that the TGA stop codon of *amyM* partially overlapped with the ATG start codon of *gfpmut3.* This resulted in the sequence *A****TG*A**, where the start codon is marked in italics, and the stop codon in bold. In this manner an out-of-frame translational fusion was created between the *amyM* and *gfpmut3* genes. For this purpose, the complete expression vector pDAM carrying *amyM* under the transcriptional control of *P(amyQ)*-*M* was amplified using oligonucleotides M gfp fw and M gfp rv3, and the *gfpmut3* gene was amplified using gfp OV fw 3 and gfp OV rv2. Complementary sequences were inserted at the 5´- and 3´-ends of the amplified fragments, which were fused by Gibson Assembly after purification via gel extraction. The resulting amplicon was used to transform *B. subtilis* DB104, resulting in plasmid pDAamyM-gfp.

### Cultivation of *B. subtilis* using the live cell array (LCA) system

*B. subtilis* strains were grown overnight in 100 µl LB medium supplemented with appropriate concentrations of antibiotics in 96-well plates at 37 °C under vigorous shaking at 1000 rpm in a Thermo Shaker L079-100 (Kisker). An over-day culture in 96-well plates was inoculated with the over-night culture at a 1/20 dilution and grown under the same conditions for 6 h. Cultivation of *B. subtilis* was carried out in a 96-well plate with flat bottom (Greiner bio-one, Cellstar) in a final volume of 100 µl of LB or EnPressoB medium (BioSilta), inoculated with 5 µl from the over-day culture. To avoid evaporation all plates were covered with a lid, incubated at 37 °C with constant shaking (fast mode: 1140 rpm) using a Biotek Synergy two multimode microplate reader. Fluorescence (excitation 485/20 mm, emission 528/20 mm) and optical density (OD_600_) were measured at 10 min intervals. For light path length correction, the OD_977_ and OD_900_ were measured and were calculated for a sample length of 1 cm by (OD_977_–OD_900_)/0.18 as described previously [34].

GFP-expression levels were corrected for background fluorescence by subtracting the fluorescence values derived from the cultivation of the wild-type strain 168 (cultivated in six independent wells). Next, normalization of the fluorescence increase per time and growth rate was calculated using the following equation: (dGFP/dt/OD_600_).

### Fluorescence microscopy

*Bacillus* cells carrying out-of-frame translational fusions of *amyM* and *gfpmut3* were grown in 20 ml LB or in EnPressoB medium in 250 ml shake flasks under continuous shaking at 200 rpm. At the chosen time points (5.5, 8, 24 and 32 h), cells were spotted on poly-l-lysin-coated microscopy slides. Fluorescence microscopy was performed with a Leica DM5500 B microscope equipped with a Leica EL6000 camera.

### Time-lapse microscopy

Time-lapse fluorescence microscopy was performed with a Leica DM5500 B microscope equipped with a motorized stage and temperature-controlled incubation chamber set at 37 °C. Movies were recorded as described previously [34]. A preculture in 25 % LB medium was made from a LB over-night culture. After reaching an OD_600_ of ~0.2 cells were applied to 25 % LB agarose (1.5 %) slides, which were prepared as described previously [37].

### Flow cytometry

*B. subtilis* strains were grown in 20 ml LB medium or EnPressoB medium in 250 ml shake flasks with baffles at 37 °C and shaking at 200 rpm. Samples were taken after 5.5, 8, 24 and 32 h of incubation and were measured via flow cytometry with a BD Accuri C6 (BD Biosciences) as described previously [25].

### SDS-PAGE

*B. subtilis* strains were grown in 20 ml LB or in EnPressoB medium in 250 ml shake flasks under continuous shaking (250 rpm, 37 °C). Growth medium fractions were separated from the cells by centrifugation (3500*g*, 15 min, 4 °C) after cultivation times of 5.5, 8, 24, and 32 h. Electrophoresis on 4–12 % Bis–Tris-NuPAGE gels (Invitrogen) was performed as described by the manufacturer’s protocol.

## Results

### Activation of the protein secretion stress response in *B. subtilis* cells hyper-secreting the α-amylase AmyM

To monitor the induction of a possible protein secretion stress response in *B. subtilis* 168 during overproduction of the α-amylase AmyM in comparison to the previously investigated α-amylase AmyQ, the expression vectors pDAM (*amyM*) or pKTH10 (*amyQ*) were used to transform *B. subtilis* 168 Hm C5. AmyQ was used as a control, because it was previously shown that its high-level expression from plasmid pKTH10 [33] causes strong induction of the P(*htrA/htrB*) promoters [19]. Importantly, strain 168 Hm C5 contained a transcriptional fusion of the *P(htrB)* promoter with the *gfpmut3* reporter gene [35]. To construct this fusion, the promoter region of *htrB* (~300 bp) plus an optimized ribosome-binding site (RBS) were fused directly upstream of the *gfpmut3* gene, and the resulting construct was integrated into the genome by single cross-over recombination. Of note, the *gfpmut3* gene codes for a GFP variant of high stability with an estimated half-life of ~10 h in *B. subtilis* that makes it a useful marker for promoter activity determination [34]. Furthermore, to study secretion stress induction, all strains were grown in two different media, namely LB and EnPressoB. The EnPressoB medium permits increased volumetric productivity for recombinant proteins in small culture volumes (e.g. ~100 µl in 96-well plates). It is based on a soluble polysaccharide in the medium from which glucose is slowly released through the action of a specific endo-glucanase [32]. In the LCA setup, the resulting slow-release glucose feeding regime based on completely soluble medium components allows the online measurement of both OD_600_ and GFP fluorescence under conditions that mimic production scale conditions.

**Fig. 1:**
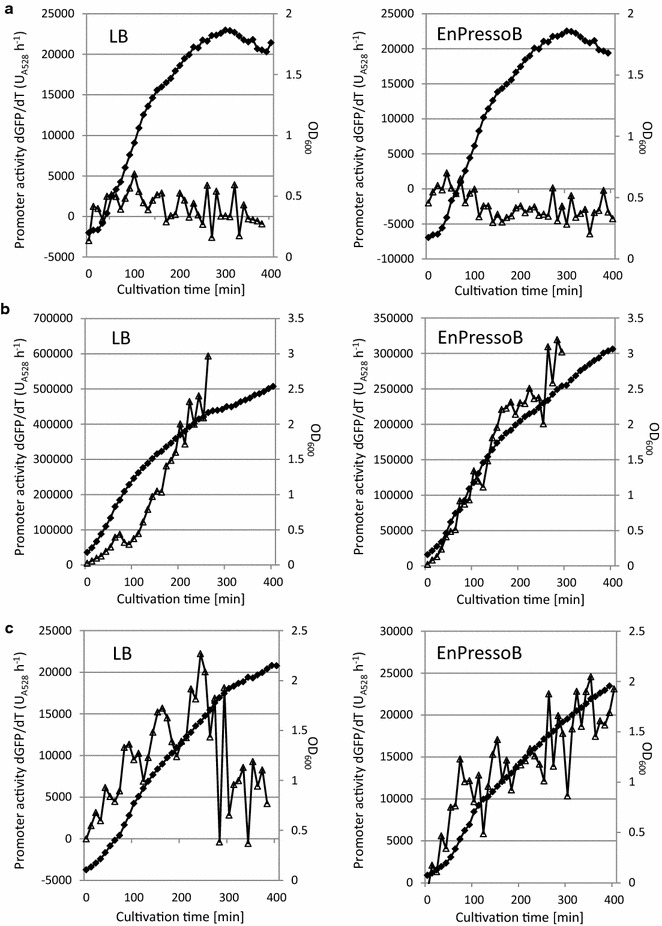
*P(htrB)* promoter activity in AmyQ- and AmyM-secreting *B. subtilis* cells. Growth profiles (*filled diamonds*) and *P(htrB)* promoter activities (*open triangles*) of *B. subtilis* strain 168 Hm C5 (**a**) and transformants carrying plasmid pKTH10 (**b**) or pDAM (**c**) were monitored by the LCA system during growth in LB and EnPressoB medium over a cultivation time of 400 min. *P(htrB)* promoter activity in the pKTH10-carrying strain yielded fluorescence intensities outside the detection range after 260 min when cells were grown in LB medium, and after 290 min when cells were grown in EnPressoB medium

The growth profiles and *P(htrB)* promoter activities of *B. subtilis* 168 HmB C5 and transformants of this strain carrying plasmids pKTH10 or pDAM upon cultivation in LB or EnPressoB medium are shown in Fig. 1. As shown in Fig. 1a, the *P(htrB)*-*gfp* fusion was not expressed in *B. subtilis* 168 HmB C5 without the α-amylase-encoding plasmids. Consistent with previous findings [16, 19], the pKTH10-carrying strain showed very high *P(htrB)* promoter activities, indicating high induction of the secretion stress response by AmyQ production both in LB and EnPressoB medium (Fig. 1b). In fact, the fluorescence intensity went outside the detection range upon growth in LB medium after ~260 min of cultivation and in EnPressoB medium after ~290 min. Although the promoter activity of *P(htrB)* in AmyQ-producing cells grown in LB medium may appear to be slightly higher than in cells grown in EnPressoB, the *P(htrB)* activities under both conditions are actually comparable when normalized to the OD_600_ (data not shown).

In contrast to AmyQ-producing cells, we found that cells producing AmyM displayed only a moderate stress response. In fact, the *P(htrB)* promoter activity in *B. subtilis* 168 Hm C5 with pDAM was ~10-fold lower than in *B. subtilis* 168 Hm C5 with pKTH10 (Fig. 1b, c). Further, slightly higher *P(htrB)* activities were measured in cells grown in EnPressoB than in cells grown in LB. Lastly, *P(htrB)* activity declined after 300 min of cultivation in LB medium, whereas the *P(htrB)* activity increased continuously upon cultivation in EnPressoB. The latter observations suggest that AmyM production was somewhat higher in EnPressoB than in LB cultures (Fig. 1c).

### Heterogeneity of protein secretion stress in AmyM-secreting *B. subtilis* cells

**Fig. 2:**
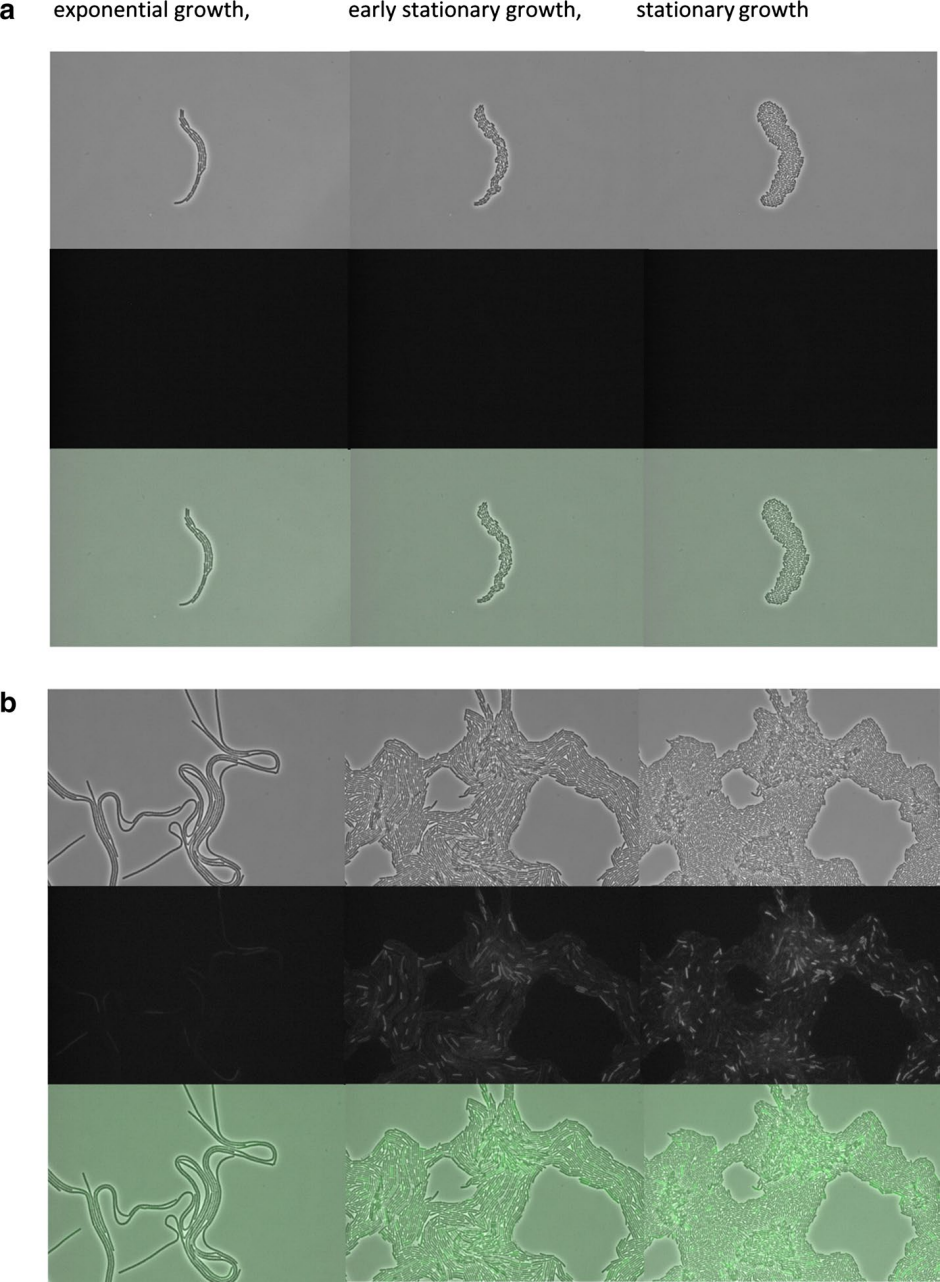
Heterogeneity of protein secretion stress in *amyM*-expressing cells. Time-lapse microscopy was performed of *B. subtilis* 168 Hm C5 (**a**) and 168 Hm C5 carrying plasmid pDAM coding for the secretory α-amylase AmyM (**b**). Strains were grown on 1.5 % agarose slides containing 25 % LB broth. Microscopy images are shown from cultures in the exponential, early stationary and stationary growth stages

To investigate the promoter *P(htrB)* activity with regard to heterogeneous or homogeneous activity in cells expressing AmyM, strain168 Hm C5::pDAM was analyzed by time-lapse microscopy using strain 168 Hm C5 as a control. Interestingly, the activity of *P(htrB)* in the AmyM-producing strain was highly heterogeneous as reflected by the differential GFP fluorescence of individual cells (Fig. 2b). In fact, two sub-populations were detected with a minor population displaying very high *P(htrB)* activity and a majority of cells with low *P(htrB)* activity (Fig. 2b). Since the observed heterogeneity in *P(htrB)* activity suggested the presence of sub-populations of high- and low-level AmyM-producing cells, the former suffering from high levels of secretion stress while the latter were only moderately stressed, we decided to further investigate the possible causes for the observed heterogeneity.

### Heterogeneity and homogeneity of *P(amyQ)*-*M* activity is growth condition-dependent

**Fig. 3:**
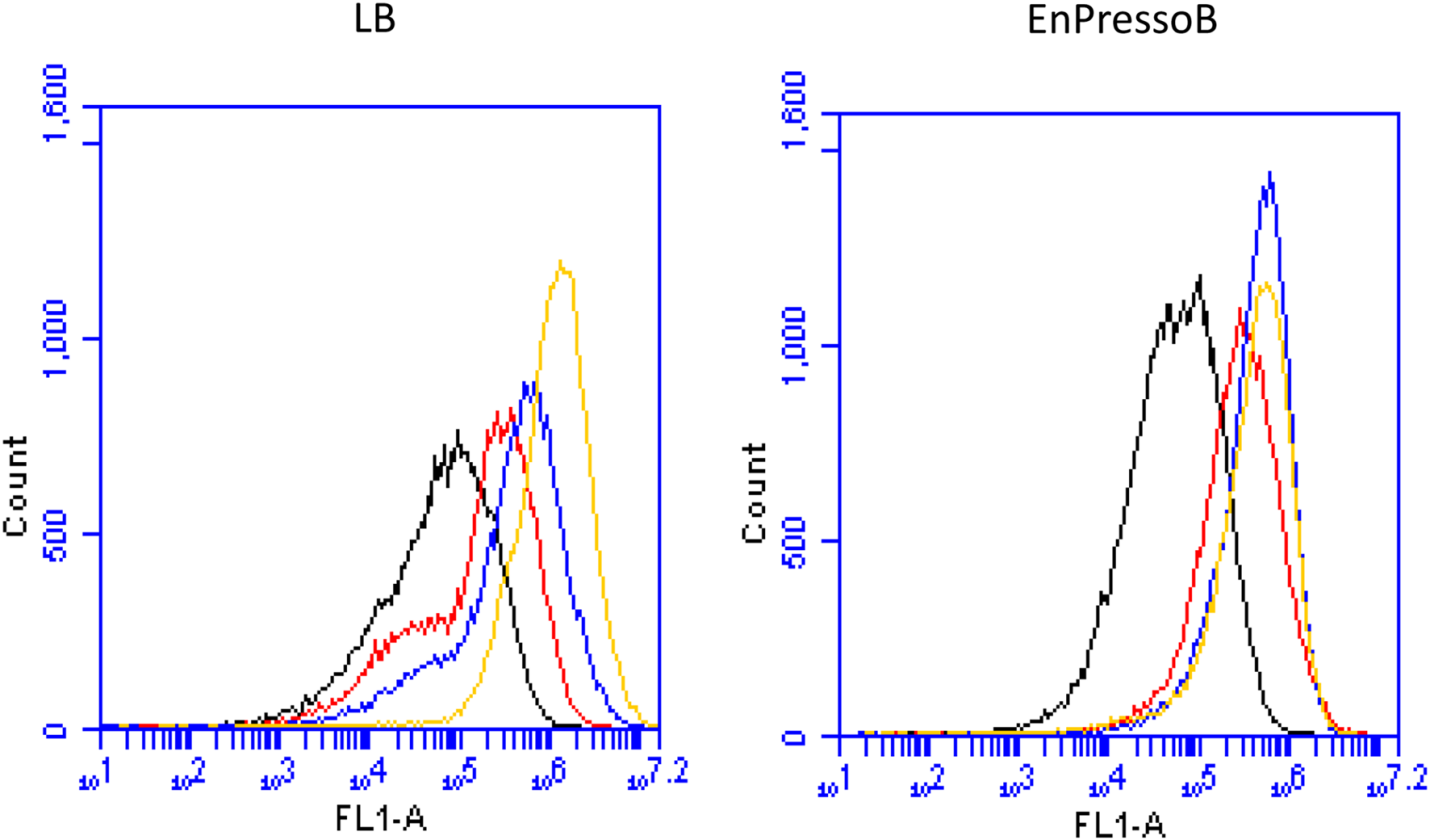
Heterogeneous and homogeneous activity of *P(amyQ)*-*M* under different growth conditions. Flow cytometry histograms are shown for *B. subtilis* strain 168::pDAamy-gfp tet carrying transcriptional fusions of *P(amyQ)*-*M*-*gfpmut3* grown in LB or EnPressoB medium. Samples were taken after 5.5 (*black*), 8 (*red*), 24 (*blue*) and 32 h (*yellow*) of cultivation time

Transcription of the *amyM* gene on pDAM is directed by the highly active *P(amyQ)*-*M* promoter. Accordingly, a possible cause for the heterogeneous induction of protein secretion stress in strain 168 Hm C5::pDAM was heterogeneity in *P(amyQ)*-*M* promoter activity, which would in turn result in heterogeneous production of AmyM. To evaluate whether promoter activity driving *amyM* expression was correlated with secretion stress, a transcriptional *P(amyQ)*-*M*-*gfpmut3* fusion was constructed and expressed from the same plasmid backbone that had been used to construct pDAM. The resulting plasmid, which also carried a tetracyclin resistance marker, was named pDAPamy-gfp tet. To investigate the possible heterogeneous activity of *P(amyQ)*-*M*, *B. subtilis* 168::pDAPamy-gfp tet was grown in LB medium or EnPressoB, and the expression of GFP in individual cells was measured using flow cytometry. As shown in Fig. 3, GFP expression was markedly heterogeneous when the cells were cultivated in LB medium. In fact, a bimodal fluorescence distribution was observed in the exponential (5.5 h), early stationary (8 h) and stationary (24 h) growth phases (Fig. 3, left panel). Only at the latest time point (32 h), a mostly homogeneous GFP expression was observed. These findings are consistent with the heterogeneous secretion stress response observed via time-lapse microscopy for cells grown on LB (Fig. 2b). Interestingly, when cells were grown on EnPressoB (Fig. 3, right panel), a unimodal fluorescence distribution was already observed in the exponential growth phase (5.5 h) with most significant differences in fluorescence distribution after 8 and 24 h of cultivation when compared to LB medium. This showed that the growth conditions imposed by the EnPressoB medium resulted in a homogenous *P(amyQ)*-*M* promoter activity.

### Homogeneous activity of the *P(amyQ)*-*M* promoter depends on DegU-P levels and growth conditions

**Fig. 4:**
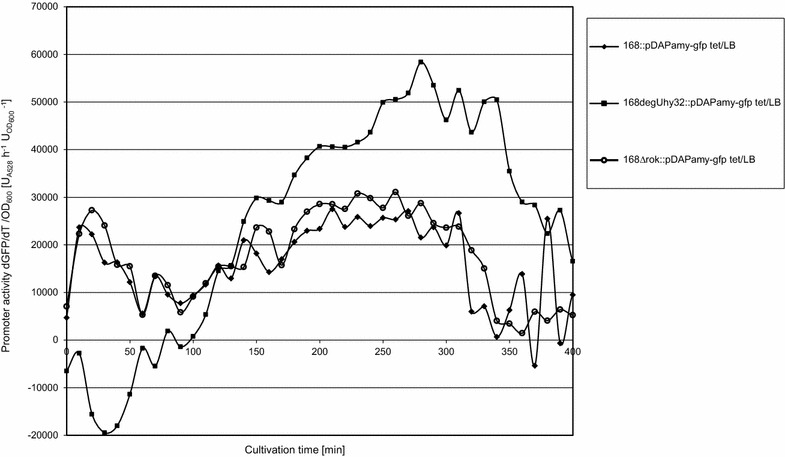
Influence of *degUhy*32 and *rok* on *P(amyQ)*-*M* promoter activity. *P(amyQ)*-*M* promoter activities were determined by fluorescence intensity measurements using LCA for the *B. subtilis* 168 wild-type strain (*diamond*) and mutants 168*degUhy*32 (*square*) or 168∆*rok* (*open circle*) carrying plasmid pDAPamy-gfp tet with the transcriptional *P(amyQ)*-*M*-*gfpmut3* fusion. Strains were grown in LB medium over a cultivation period of 400 min

The two-component regulatory system DegS-DegU is known to regulate the synthesis of many secretory enzymes [38]. In addition, DegU is a master regulator for cell differentiation [39, 40]. The so-called *degUhy*32 mutation in the *degU* gene is known to increase the half-life of the phosphorylated form of DegU, leading to hyper-production of different enzymes [41, 42]. Furthermore, it was previously demonstrated for the *P(aprE)* promoter that the DegU-P levels correlate with noisy transcription of *aprE* [43]. Similar to *P(aprE)*, *P(amyQ)*-*M* contains a DegU-binding motif (http://dbtbs.hgc.jp/). Rok is another regulator that is known to modulate the expression of membrane-localized and secreted proteins [44]. We therefore decided to analyze the influence of DegU-P and Rok on *P(amyQ)*-*M* activity. For this purpose, the transcriptional *P(amyQ)*-*M*-*gfpmut3* fusion was introduced in the *B. subtilis* strains 168*degUhy*32 and 168Δ*rok*; the former strain carries the *degUhy*32 allele, while the *rok* gene was deleted from the latter strain. To measure the influence of these mutations on overall *P(amyQ)*-*M* activity, the respective transformants carrying pDAPamy-gfp tet were grown in LB for LCA analysis. As expected, the 168*degUhy*32 variant showed increased promoter activity of *P(amyQ)*-*M* (Fig. 4). In contrast, the 168Δ*rok* strain showed similar promoter activity levels as the 168 wild-type (Fig. 4).

**Fig. 5:**
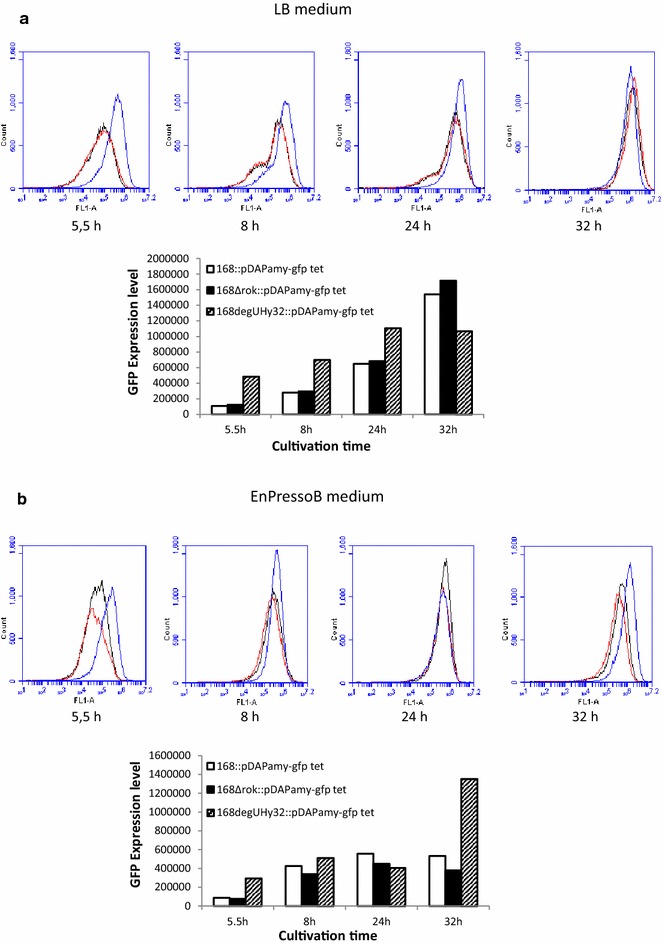
Influence of *degUhy*32 and *rok* mutations on heterogeneous activity of *P(amyQ)*-*M* under different growth conditions. GFP expression levels and flow cytometry histograms are shown for *B. subtilis* strains 168 (*black*), 168*degUhy*32 (*blue*) and 168*Δrok* (*red*) carrying plasmid pDAPamy-gfp tet with the transcriptional *P(amyQ)*-*M*-*gfpmut3* fusion upon growth in LB (**a**) or EnPressoB (**b**) medium. Samples were taken after 5.5, 8, 24 and 32 h of cultivation

To investigate whether the increased *P(amyQ)*-*M* activity in cells bearing the *degUhy*32 allele might be due to reduced levels of expression heterogeneity, we performed a flow cytometric analysis of GFP expression by individual cells grown in LB (Fig. 5a). Consistent with the LCA measurements in Fig. 4, the flow cytometry data showed a clear shift to higher GFP expression levels for strain 168*degUhy*32 as compared to the wild-type strain 168. Specifically, the 168*degUhy*32 strain displayed ~4.5-fold higher GFP fluorescence during exponential growth (5.5 h) and ~2.5-fold higher after 8 h of cultivation (Fig. 5a, bottom). At the same time, the bimodal GFP fluorescence distribution observed in the wild-type strain after 8–24 h of growth was shifted to a unimodal GFP fluorescence distribution in the 168*degUhy*32 strain (Fig. 5a, top). This implies a more homogeneous *P(amyQ)*-*M* activity in the 168*degUhy*32 strain. In contrast, the 168Δ*rok* strain showed no changes in the overall GFP expression levels and the distribution of GFP fluorescence over individual cells as compared to the wild-type strain 168 (Fig. 5a, top). These findings are consistent with the LCA data for the 168Δ*rok* strain as shown in Fig. 4.

When grown on EnPressoB medium, the 168 wild-type strain showed a more homogeneous activity of *P(amyQ)*-*M* as represented by a unimodal GFP fluorescence distribution when compared to cells grown in LB medium (Fig. 5b, top). This is consistent with the results shown in Fig. 3. Notably, also the 168*degUhy*32 and 168Δ*rok* strains showed the same unimodal fluorescence distribution as the 168 wild-type strain when the cells were grown in EnPressoB (Fig. 5b, top). This implies that growth on EnPressoB leads to a homogeneous *P(amyQ)*-*M* promoter activity. The only significant difference was that the GFP expression levels were higher in the 168*degUhy*32 strain during the exponential phase and in the late stationary growth phase compared to the wild-type and the 168Δ*rok* strains. Specifically, the detected GFP expression levels in the 168*degUhy*32 strain were 3.5-fold higher after 5.5 h, and 2.5-fold higher after 32 h of cultivation, than in the wild-type strain (Fig. 5b, bottom). Together, the present analyses imply that both increased levels of phosphorylated DegU-P and the slow-release glucose feeding regime during cultivation on EnPressoB medium result in a more homogeneous *P(amyQ)*-*M* promoter activity.

### Homogeneous and heterogeneous translation of AmyM

**Fig. 6:**
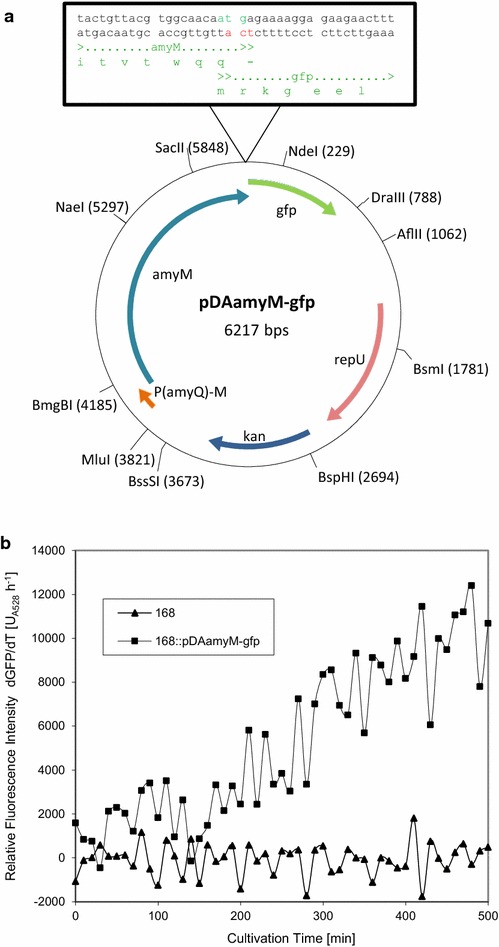
Out-of-frame translational *amyM*-*gfpmut3* fusion: Plasmid map of pDAamyM-gfp and functional translation of GFP. The plasmid map of pDAamyM-gfp represents a high-copy plasmid for expression of α-amylases carrying a translationally coupled out-of-frame fusion of *amyM* and *gfpmut3.* pDAamyM-gfp plasmid ORFs are indicated by *thick colored arrows*, with the direction of transcription and the identity of each ORF indicated. Relevant restriction sites are also indicated. Sequence details of the *amyM*-*gfpmut3* fusion are shown in the *box* (**a**). LCA measurements with *B. subtilis* 168::pDAamyM-gfp show the functional translation of GFP. The increase of fluorescence intensity over time [dGFP/dT] was monitored using the LCA system with *B. subtilis* 168 and the 168 strain carrying plasmid pDAamyM-gfp during cultivation in EnPressoB medium (**b**)

**Fig. 7:**
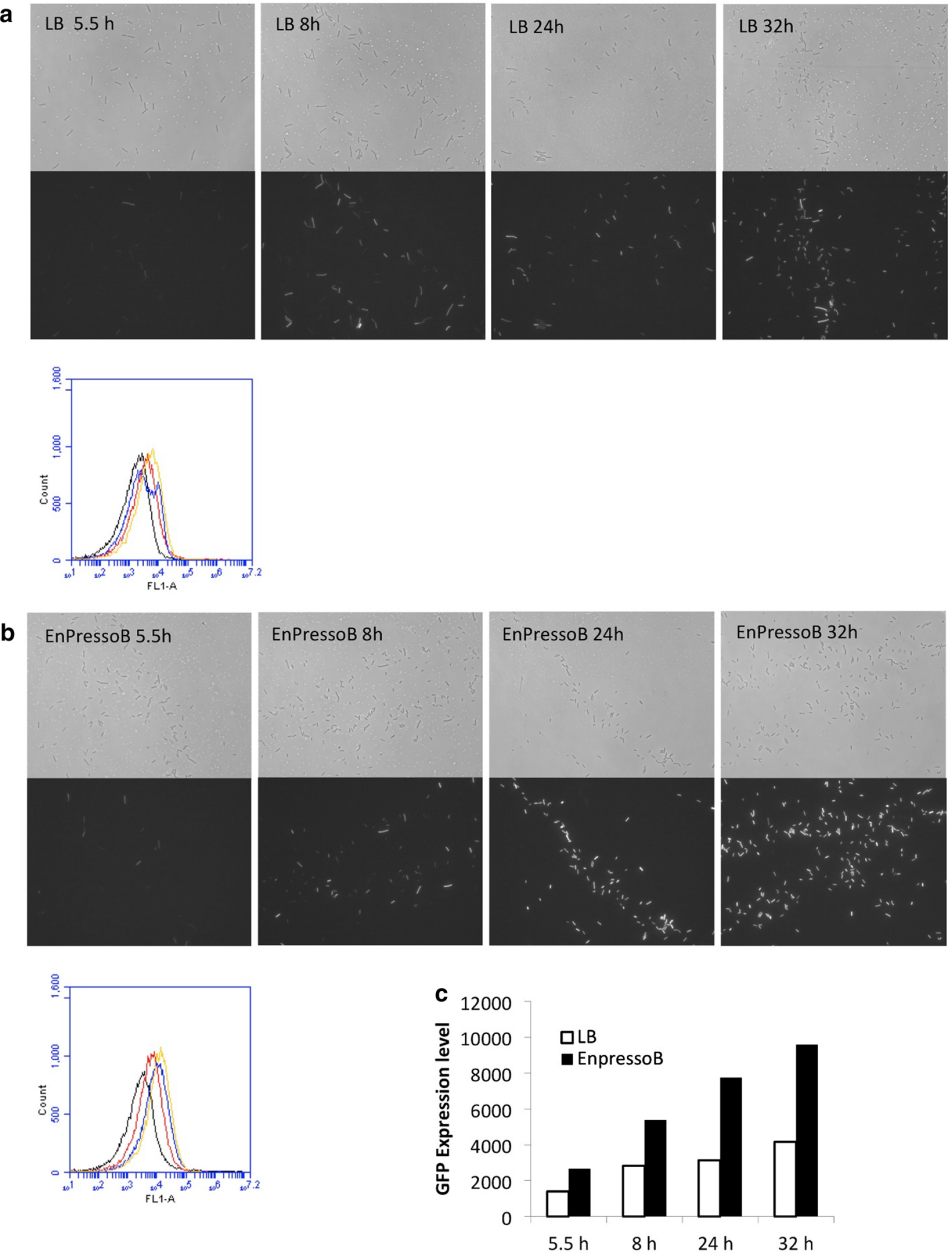
Fluorescence microscopy and flow cytometric analysis of GFP expression in *B. subtilis* 168::pDAamyM-gfp. Fluorescence microscopy and flow cytometry were performed with *B. subtilis* strain 168 carrying plasmid pDAamyM-gfp that encodes the out-of-frame translational *amyM*-*gfpmut3* fusion upon cultivation in LB (**a**) or EnPressoB (**b**) medium. Cells were analysed by microscopy (**a**, **b**, *top*) and flow cytometry after 5.5 (*black*), 8 (*red*), 24 (*blue*), and 32 h (*yellow*), and histograms of GFP expression are shown (**a**, **b**, *bottom*). A comparison of GFP fluorescence levels detected upon growth in LB medium and EnPressoB is shown in **c**

**Fig. 8:**
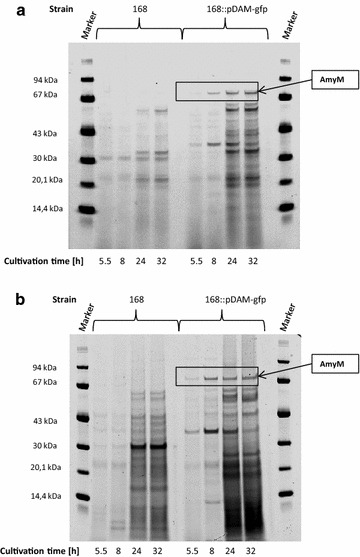
Production and secretion of AmyM in *B. subtilis* 168 carrying the out-of-frame translational *amyM*-*gfpmut3* fusion. *B. subtilis* strains 168 and 168 carrying pDAamyM-gfp were grown in LB (**a**) or EnPressoB medium (**b**). SDS PAGE was performed with culture supernatant samples after cultivation times of 5.5, 8, 24 and 32 h

A relevant question triggered by the observed heterogeneity in the protein secretion stress response and the *P(amyQ)*-*M* promoter activity was whether this heterogeneity could also be detected in the translation of AmyM, which would provide further evidence for AmyM production heterogeneity. For real-time monitoring of the translation of AmyM, we designed an out-of-frame translational fusion between *amyM* and *gfpmut3*. This was necessary, because in-frame fusions between secretory proteins and GFP cannot be effectively translocated across the membrane via the Sec pathway [45, 46]. The out-of-frame translational fusion was generated by creating an overlap between the stop codon of *amyM* and the start codon of *gfpmut3* (Fig. 6a). In this setting, the translation of GFP is dependent on the efficient translation of AmyM, because there is no SD sequence upstream of the GFPmut3-coding region. Further, the expression of this out-of-frame translational *amyM*-*gfpmut3* fusion was directed from *P(amyQ)*-*M* using the same plasmid backbone as in the above experiments. The resulting plasmid named pDAamyM-gfp is depicted in Fig. 6a. LCA analyses with *B. subtilis* 168::pDAamyM-gfp showed an increasing fluorescence intensity during cultivation, which confirmed the effective translation of GFP (Fig. 6b). This was further evidenced by fluorescence microscopy and flow cytometry, which also allowed us to monitor homogeneity or heterogeneity in the coupled transcription-translation of the *amyM*-*gfpmut3* fusion upon growth in LB or EnPressoB medium (Fig. 7a, b). The results show that the GFP fluorescence distribution amongst cells grown in the EnPressoB medium was more homogeneous than that of cells grown in LB (Fig. 7, compare the lower panels in a, b). Moreover, the GFP fluorescence levels were ~2-fold higher upon growth in EnPressoB medium in comparison to growth in LB (Fig. 7c). To verify whether this higher GFP expression level reflects also the translation and subsequent secretion of AmyM into the culture broth, growth medium fractions were analysed by SDS-PAGE. Indeed, the secretion of AmyM by the 168::pDAamyM-gfp strain grown in EnPressoB (Fig. 8b) started at an earlier time point and reached higher levels than when these cells were grown in LB (Fig. 8a). As expected, no AmyM was detectable in the growth medium of the wild-type strain 168 (Fig. 8). Together, these observations show that the homogeneous or heterogeneous expression of *amyM*, as driven by the *P(amyQ)*-*M* promoter under different growth conditions, is reflected in the homogeneous or heterogeneous translation of GFP when using an out-of-frame translational fusion between *amyM* and *gfpmut3*.

## Discussion

The present study was initially aimed at investigating the possible protein secretion stress response of *B. subtilis* 168 upon high-level expression of the heterologous α-amylase AmyM from *G. stearothermopilus*. Our results showed that AmyM production only led to a relatively low activation of the secretion stress-responsive *P(htrB)* promoter, in contrast to the production of the α-amylase AmyQ from *B. amyloliquefaciens*, which caused strong *P(htrB)* activation in accordance with previously published data [16, 19]. Intriguingly, the induction of *P(htrB)* in AmyM producing cells was “noisy” when cells were grown in LB medium, suggesting the presence of sub-populations of low-level and high-level AmyM-producing cells. Since low-level producing cells are unwanted in industrial fermentations, this finding was followed up by investigating the effects of a slow-release glucose feeding regime mimicking the industrial setting and particular mutations in DegU and Rok, two major regulators of secretory protein production. This showed that slow-release glucose feeding and the *degUhy*32 mutation can both lead to homogeneous high-level production of AmyM in *B. subtilis* 168.

The pDAM plasmid that was used for AmyM production carries the *amyM* gene under the transcriptional control of the *P(amyQ)*-*M* promoter. This promoter is a slightly modified version of the *amyQ* promoter, which was developed for industrial-scale fermentations. Our present results show that this promoter is “noisy” when cells are cultured on LB medium. In turn this leads to noisy AmyM production which, most likely, explains the heterogeneous secretion stress response. To date, this phenomenon had not been described. Yet, it is a very useful observation because it implies that heterogeneous expression of a secreted target protein can be detected by assessment of heterogeneity in the secretion stress response with transcriptional *P(htrB)*-*gfp* fusions.

Gene expression heterogeneity is a general biological phenomenon that allows bacterial populations to rapidly adapt to changing environmental conditions [47]. As a resident of the soil and plant rhizosphere, *B. subtilis* is a master of adaptation and, accordingly, it is not surprising that it commits itself to differentiation into a multitude of cell types. These differentiation processes encompass motility, biofilm formation, development of genetic competence for DNA binding and uptake, sporulation, and also the production of secreted degradative enzymes [22–25, 48, 49]. The production of secretory enzymes in industrial-scale fermentations is aimed towards achieving homogenous populations of highly productive cells, especially during stationary growth [21, 43, 50]. Therefore, promoters to be used in the high-level gene expression for industrial purposes should ideally be strong and controllable. Such promoters can be classified as inducer-specific, growth phase-specific, stress-specific or auto-inducible [50]. This relates to the fact that strategies for large-scale high-yield fermentations for protein production are often based on a first stage where high cell densities are generated without stressing the cells, and a subsequent protein production phase during which the target gene is stably expressed at high-level over a long period of time and preferably in a homogeneous manner. Promoters satisfying these requirements are for example the Isopropyl β-d-1-thiogalactopyranoside (IPTG)-inducible *P(spac)* promoter or the xylose-inducible *P(xynA)* promoter. These promoters, however, have the disadvantage that the addition of IPTG or xylose during the fermentation process is relatively costly [2, 50]. Examples of growth phase-regulated promoters induced during the stationary phase are the *P(amyE)* and *P(aprE)* promoters of *B. subtilis* which, respectively, direct expression of the α-amylase AmyE and the serine protease AprE (i.e. subtilisin). Both promoters are highly threshold-dependent on specific transcriptional regulators [50, 51]. Our present studies with transcriptional *gfp* fusions indicate that this is also true for the *P(amyQ)*-*M* promoter, because the highest levels of GFP expression were detected during late growth stages, and because the *degUhy*32 mutation strongly enhanced GFP expression. In this respect it is noteworthy that the high-yield production of native secreted enzymes of *B. subtilis*, such as proteases, α-amylase and levansucrase, is significantly dependent on the DegS-DegU two-component system [38]. Several mutations in *degS* and *degU* that lead to overproduction of proteases have been characterized previously [52, 53]. One of these is the *degUhy*32 mutation, which increases the half-life of phosphorylated DegU [42]. Accordingly, our present results obtained with the *degUhy*32 strain suggest that high levels of phosphorylated DegU lead to a more homogeneous and enhanced *P(amyQ)*-*M* activity. Consistent with the enhanced activity of *P(amyQ)*-*M* in the *degUhy*32 mutant, this promoter contains a DegU binding motif (http://dbtbs.hgc.jp/). These findings are, thus, fully in line with previous studies, which showed that noisy activity of the DegU-dependent *P(aprE)* promoter is also correlated with the levels of phosphorylated DegU [43, 54].

Batch feeding of catabolite-repressing carbon sources is a well-known approach to reach high product levels for target proteins that are subject to carbon catabolite repression, such as the α-amylases of *B. licheniformis* and *B. amyloliquefaciens* [2]. To simulate the industrial carbon-limited conditions in a scaled-down setting, we employed the EnPressoB medium, since this medium simulates a continuous, rationally limited glucose feeding [32]. Our finding that this slow-release glucose feeding also leads to more homogeneous activity of *P(amyQ)*-*M* is unprecedented. While it is well known that medium optimization is crucial for high-yield protein production [55, 56], it was not yet known that this optimization may, at least partially, relate to the fact that a more homogeneous population of high-producing cells is obtained. It will be interesting to verify in future studies whether this shift to a more homogeneous expression pattern also applies to other proteins of commercial interest that are expressed from different promoters.

To monitor the homogeneous or heterogeneous translation of *amyM* in *B. subtilis*, we employed an out-of-frame translationally coupled *amyM*-*gfp* fusion where the stop codon of *amyM* overlapped with the start codon of the *gfp* gene. This type of translational coupling occurs also naturally in *B. subtilis* 168, for example in the *gerAA*-*gerAB*-*gerAC* Operon [57]. In our *amyM*-*gfp* fusion, we avoided the presence of a spacer between the two genes and the presence of a RBS in the proximity of the *gfp* start codon as they are present in other *gfp* operon-like expression constructs that were previously published [58, 59]. Importantly, this approach allowed us to monitor *amyM* translation by measuring the intracellular appearance of GFP fluorescence, without interfering with the secretion of the co-translated AmyM. Compared to the classical in-frame GFP fusion technology [59, 60], an additional advantage of out-of-frame translationally coupled GFP fusions is that they circumvent the frequently observed instability of large GFP fusion proteins. We conclude from the results of our studies that the transcriptional and out-of-frame translational *gfp* fusion constructs as described here represent a highly effective tool kit for monitoring potential bottlenecks in high-yield protein production by pinpointing potentially limiting steps in transcription, translation and/or protein secretion [61]. Additionally, the out-of-frame translational fusion of genes for secretory proteins with *gfp* may serve as a tool for assessing target protein degradation by the different proteases produced by *B. subtilis* [62]. Although, this potential problem was not specifically addressed in the present study, it is known that *B. subtilis* 168 secretes a cocktail of eight proteases (i.e. AprE, Bpr, Epr, Mpr, NprB, NprE, Vpr and WprA) that may degrade other cellular and secreted proteins [62–64]. In addition, secretion-stressed cells overproduce the quality control proteases HtrA and HtrB, which may add to the loss of product. Especially in those cases where low production levels are observed, out-of-frame translational *gfp* fusions are attractive tools to distinguish between production bottlenecks at the translational and post-translational levels. Eventually, such tools may even be implemented for the on-line monitoring of protein production during large-scale fermentation processes using state-of-the-art GFP sensors [65–67].

## Conclusion

In the present study, we have employed different transcriptional and translational *gfp* fusions to assess the production of the α-amylase AmyM in *B. subtilis* in real time. Importantly, such fusions allowed us both to monitor the cellular secretion stress response, the expression of *amyM* and the homogeneity of the AmyM-producing cell population. We conclude that, together, the followed approaches represent a highly effective pipeline for optimizing transcription, translation and overall expression homogeneity during production strain and process development.

## Availability of data and material

The datasets supporting the conclusions of this article are included in the article.
